# Phogrin Regulates High-Fat Diet-Induced Compensatory Pancreatic β-Cell Growth by Switching Binding Partners

**DOI:** 10.3390/nu16010169

**Published:** 2024-01-04

**Authors:** Chisato Kubota, Ryoko Torii, Masahiro Hosaka, Toshiyuki Takeuchi, Hiroshi Gomi, Seiji Torii

**Affiliations:** 1 Institute for Molecular and Cellular Regulation, Gunma University, Maebashi 371-8512, Gunma, Japantstake@gunma-u.ac.jp (T.T.); 2 Department of Nutrition, Takasaki University of Health and Welfare, Takasaki 370-0033, Gunma, Japan; 3 Department of Biotechnology, Akita Prefectural University, Akita 010-0195, Akita, Japan; mhosaka@akita-pu.ac.jp; 4 Department of Veterinary Anatomy, College of Bioresource Sciences, Nihon University, Fujisawa 252-0880, Kanagawa, Japan; gomi.hiroshi@nihon-u.ac.jp; 5 Center for Food Science and Wellness, Gunma University, Maebashi 371-8511, Gunma, Japan

**Keywords:** high-fat diet, insulin receptor substrate, insulin signaling, islet antigen, pancreatic β-cell mass, secretory granules

## Abstract

The receptor protein tyrosine phosphatase phogrin primarily localizes to hormone secretory granules in neuroendocrine cells. Concurrent with glucose-stimulated insulin secretion, phogrin translocates to pancreatic β-cell plasma membranes, where it interacts with insulin receptors (IRs) to stabilize insulin receptor substrate 2 (IRS2) that, in turn, contributes to glucose-responsive β-cell growth. Pancreatic β-cell development was not altered in β-cell-specific, phogrin-deficient mice, but the thymidine incorporation rate decreased in phogrin-deficient islets with a moderate reduction in IRS2 protein expression. In this study, we analyzed the β-cell response to high-fat diet stress and found that the compensatory expansion in β-cell mass was significantly suppressed in phogrin-deficient mice. Phogrin–IR interactions occurred only in high-fat diet murine islets and proliferating β-cell lines, whereas they were inhibited by the intercellular binding of surface phogrin under confluent cell culture conditions. Thus, phogrin could regulate glucose-stimulated compensatory β-cell growth by changing its binding partner from another β-cell phogrin to IR in the same β-cells.

## 1. Introduction

Phogrin (IA-2β or PTPRN2) and IA-2 (PTPRN or ICA512), autoantigens in insulin-dependent diabetes, are type 1 transmembrane proteins belonging to the IA-2 family of protein-tyrosine phosphatase (PTP) [1,2,3,4,5,6]. Both proteins share the highest sequence similarity in the cytoplasmic PTP domain and lack phosphatase activity for common PTP substrates due to amino acid mutations in the evolutionarily conserved catalytic domain [7,8]. In contrast, phogrin has limited activity towards phosphatidylinositol, although its relationship to physiological function remains unknown [9]. Phogrin and IA-2 are expressed in various neuroendocrine cells including the pancreatic islets, anterior pituitary, gastrointestinal tract, and neurons and are specifically targeted and localized to the hormone-containing secretory granules (SGs) in these cells [1,5,10,11,12].

Pancreatic β-cells are the key components in blood glucose regulation as they secrete insulin in response to high glucose stimulation. Specific dense SGs store insulin for proper secretory function, and proteins residing in SGs are supposed to be involved in secretory processes and exocytotic functions [13,14]. Systemic phogrin or IA-2 gene deletion in mice decreases glucose-stimulated insulin secretion (GSIS); however, an in vitro perfusion experiment has shown that the secretory response to glucose in phogrin and IA-2 double-knockout islets is essentially normal compared with that in control islets [15,16,17]. A recent study has reported that the number of SGs reduced in single- and double-knockout islets [18], which may affect the decline in plasma insulin levels, although the molecular mechanisms underlying the regulation of secretory granule content remain unclear. However, specific knockdown of these genes in pancreatic β-cell lines (mouse MIN6, rat INS-1) does not affect GSIS and insulin content, whereas the cell proliferation rate substantially decreases [19,20]. Consistently, IA-2-deficient mice have low β-cell regeneration rates after partial pancreatectomy, and the replicative activity slightly decreased in islets isolated from a pancreatic β-cell-specific phogrin gene knockout murine model [8,20]. In contrast, adenovirus-mediated phogrin or IA-2 overexpression in rodent cell lines promotes their growth without changing the insulin content and GSIS [19].

In pancreatic β-cells, insulin secretion stimulated by glucose triggers autocrine- and paracrine-mediated insulin receptor (IR) activation. GSIS promotes phogrin to translocate from insulin-containing SGs to the plasma membrane, where it interacts with activated IR [19]. The transient interaction of phogrin with IR is important for downstream IRS2 signal transduction by preventing protein-tyrosine phosphatase 1B (PTP1B) oxidation. Indeed, in pancreatic β-cell-specific phogrin knockout mice, IRS2 levels in islets were reduced by 44% compared to those in control mice [8]. Furthermore, phogrin reduction by specific shRNAs in β-cell lines or isolated islets led to a gradual decrease in IRS2 protein levels, and phogrin silencing-induced IRS2 degradation depended on a PI3K- and mTOR-mediated negative feedback mechanism of glucose-induced insulin signaling [8,19]. Dephosphorylation of IR by PTP1B occurs to prevent persistent activation of downstream signals and protects the IRS2 protein from degradation. These provided data suggest that phogrin-regulated prompt activation of insulin signaling forms the basis for autocrine insulin action in pancreatic β-cells.

Glucose and glycolysis have a principal role in β-cell proliferation, and glucokinase-induced *IRS2* is involved in compensatory β-cell proliferation in response to high-fat, diet-promoted insulin resistance [21,22]. Although, how autocrine insulin signaling contributes to β-cell proliferation under any state remains unclear; multiple transgenic mice studies suggest that IRS2 is critical in β-cell hyperplasia in response to insulin resistance, in addition to regulating β-cell mass [23,24]. Clarifying such molecules and regulation mechanisms is important because inadequate compensatory β-cell proliferation may be a fundamental defect underlying type 2 diabetes development [25]. Meanwhile, studies using recently developed techniques have revealed that β-cells are heterogenous populations, and β-cells that have proliferative ability have not yet been specified within islets [26,27,28].

In this study, we demonstrate that phogrin is involved in compensatory β-cell proliferation in response to high-fat, diet-induced insulin resistance. Furthermore, we showed that the cells expressing the phogrin–IR complex have a proliferative capacity and are distinct from those expressing a homologous phogrin complex.

## 2. Materials and Methods

### 2.1. Antibodies and Reagents

The anti-phogrin and IA-2 rabbit antibodies and the murine monoclonal antibody against phogrin (C38-2) have been previously characterized [19,29]. C38-2 recognizes the luminal (extracellular) matN region of phogrin protein structure encompassing amino acids 414 to 599. The anti-5-bromo-2′-deoxyuridine (anti-BrdU) mouse and anti-IRβ rabbit antibodies were purchased from Roche Diagnostics (Indianapolis, IN, USA). The guinea pig anti-insulin antibody, anti-β-actin, and anti-GST mouse monoclonal antibodies, and the glutathione sepharose 4B were purchased from Sigma (St. Louis, MO, USA). The anti-GFP mouse and anti-phosphotyrosine mouse (pY20) antibodies were purchased from BD Biosciences (Lexington, KY, USA). Anti-IRS2 rabbit and anti-IRβ mouse antibodies were purchased from Upstate Biotechnology (Lake Placid, NY, USA) and Chemicon-Millipore (Temecula, CA, USA), respectively.

### 2.2. Animals and Insulin Assay

Pancreatic β cell-specific phogrin gene knockout (KO) and control (Ctrl) mice with a C57BL/6J genetic background have been previously described [8]. KO mice for the experiments were obtained by crossing heterozygotes. Male mice with similar body weight and age were used for all experiments. Mice had free access to water and the 5-week-old mice had normal chow (CE-2; CLEA Japan, Fujinomiya, Japan) or a high-fat, high-sucrose diet (HFD) for 20 weeks. The mice were housed at 23 °C with a 12 h light–dark cycle. The HFD provides 55%, 28%, and 17% calories from fat, carbohydrates, and protein, respectively (Oriental Yeast Co., Ltd., Tokyo, Japan). We measured blood glucose levels using a glucometer (Sanwa Kagaku, Nagoya, Japan) and plasma insulin levels using ELISA kits for mouse insulin (LBIS Insulin Mouse S-Type; Shibayagi, Shibukawa, Japan). For the glucose tolerance test, overnight fasted mice were intraperitoneally injected with 1.0 g/kg glucose, and blood samples were obtained 0, 15, 30, 60, and 120 min after the injection. For the insulin secretion assay, pancreatic islets were isolated after the injection of 500 U/mL of collagenase (type XI; Sigma) through the pancreatic duct and subsequent digestion at 37 °C for 30 min. The islets were handpicked under a dissection microscope and cultured in RPMI 1640 medium with 10% FBS for 24 h. The amounts of insulin secreted from 90 islets for 1 h were measured both under low glucose (LG: 2.2 mM) and high glucose (HG: 17.8 mM) conditions in modified Krebs–Ringer buffer (2 mM glucose, 15 mM HEPES, pH 7.4, 120 mM NaCl, 5 mM KCl, 24 mM Na_2_HCO_3_, 2 mM CaCl_2_, 1 mM MgCl_2_, and 0.1% bovine serum albumin). To evaluate insulin content in islets, insulin was extracted from the islets after a secretion assay in an acid–ethanol solution (70% ethanol and 0.18 M HCl). Total and secreted insulin were measured using ELISA. All the animal experiments were conducted in accordance with the guidelines of the Animal Care and Experimentation Committee of Gunma University.

### 2.3. Cell Lines

MIN6 cells and MIN6 stably expressing phogrin–EGFP [8] were cultured in Dulbecco’s modified Eagle’s medium supplemented with 10% fetal bovine serum. For stable cell line construction, MIN6 cells were transfected with pcDNA3–phogrin–GST [19] using Lipofectamine 3000 reagent (Invitrogen, Waltham, MA, USA) and stable clones were selected in the presence of G418. Isolated colonies of the phogrin–GST-transfected cells (a total of 12 clones) were transferred to new culture dishes for propagation and further selected via immunoblotting analysis using an anti-GST antibody. Glucose-responsive insulin secretion in stable cells was measured as previously reported [19].

### 2.4. β-Cell Mass and β-Cell Proliferation

The mice were anesthetized by intraperitoneally injecting 0.1 mg/g/bw sodium pentobarbital and then processed for immunohistochemical analyses [29]. Removed pancreases were fixed in 4% paraformaldehyde in 0.1 M phosphate buffer (pH 7.4) and embedded in paraffin. Paraffin sections (5 µm thick) were subjected to microwave antigen retrieval in boiling 0.01 M sodium citrate buffer (pH 6.0) for 5 min. These sections were blocked via incubation with 5% normal goat serum (Cat. no. 005-001-121, Jackson ImmunoResearch Laboratories, Inc., West Grove, PA, USA) in phosphate-buffered saline for 30 min before the addition of antibodies. The sections were labeled with a guinea pig anti-insulin antibody and detected using the avidin–biotin–peroxidase technique (Vector Laboratories, Newark, CA, USA), and counterstaining was performed using Mayer’s hematoxylin. The images were acquired using a microscope (Olympus BX-50, Olympus Life Science, Tokyo, Japan) equipped with a SenSys^TM^ charge-coupled device camera (Photometrics, Tucson, AZ, USA). To quantify the percentage of the pancreas area occupied by β-cells, the areas of insulin staining were measured using BZ-X700 (Keyence, Osaka, Japan) and Image J (NIH). Five sections were scored for each mouse, with four mice per group. For β-cell proliferation, the mice received 0.8 mg/mL BrdU in drinking water for 72 h. The pancreas sections were stained with anti-insulin and anti-BrdU antibodies and 4′,6-diamidino-2-phenylindole. After an overnight incubation with primary antibodies at 4 °C, sections were incubated for 2 h at room temperature with a combination of secondary antibodies: Alexa Fluor 488-conjugated anti-mouse IgG and Alexa Fluor 555-conjugated anti-guinea pig IgG, both diluted to a 1:2000 ratio (Invitrogen/Thermo Fisher Scientific, Waltham, MA, USA). The percentage of BrdU-positive nuclei per insulin-positive cells was calculated. At least 2000 β-cells were counted for each mouse.

### 2.5. Immunoprecipitation Analysis

Pancreatic islets obtained from three male mice (25 weeks old) for each diet were pooled and used for immunoprecipitation analysis. Mouse islets or MIN6 cells were extracted with lysis buffer A (20 mM Tris, pH 7.5, 150 mM NaCl, 1% Nonidet P-40, 2 mM EDTA, 0.5 mM PMSF, 5 μg/mL aprotinin, 5 μg/mL leupeptin, and 1 μg/mL pepstatin). Cell extracts were normalized for total protein content using the bicinchoninic acid method (Pierce). Protein extracts were incubated with 0.5 mg/mL anti-phogrin or 0.4 mg/mL anti-GFP antibodies, and the antibodies were collected via agitation with protein G–sepharose 4FF (Sigma-Aldrich). Immunoprecipitants and original protein extracts were separated via SDS-polyacrylamide gel electrophoresis and then transferred onto an Immobiron-P membrane (Millipore, Bedford, MA, USA). After incubation with anti-IRβ mouse antibody and horseradish peroxidase-conjugated secondary antibody (Jackson ImmunoResearch Laboratories, Inc.), the blots were developed using the emission chemiluminescence detection method with exposure to X-rays.

### 2.6. Co-Culture Analysis

MIN6/phogrin–EGFP and MIN6/phogrin–GST cells were mixed in a 1:1 ratio and cultured on 100 mm dishes for 48 h. Cells at high or low densities were extracted using lysis buffer A, and another mixture (lysate mix) was prepared from the independently cultured cell extracts. Proteins bound to phogrin–GST were collected via agitation with glutathione sepharose. The washed precipitates were analyzed via immunoblotting using an anti-GFP mouse antibody.

### 2.7. Statistical Analysis

Results are given as the mean ± standard errors of the means (SEMs), except when otherwise indicated. Differences between groups were analyzed using Student’s *t* test or the Mann–Whitney U test. *p* < 0.05 was considered statistically significant.

## 3. Results

### 3.1. Islet β-Cell Development Is Unaffected by Phogrin Deletion

**Figure 1:**
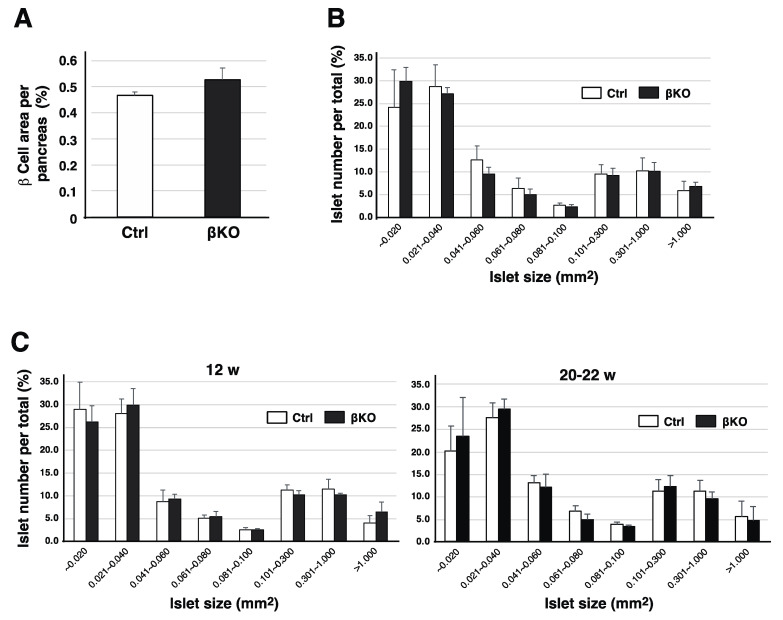
**Pancreatic β-cell mass and islet size distribution in phogrin-deficient mice:** (**A**) Immunohistochemical analyses of pancreas tissue from control (Ctrl: Cre^+/−^_Phogrin^+/+^) or knockout (βKO: Cre^+/−^_Phogrin^fl/fl^) 7-week-old male mice using anti-insulin antibody. Areas of β-cells relative to pancreas area were quantified and summed, and data are presented as means ± standard errors of the mean (SEM; *n* = 4). (**B**) Using the data (**A**), the number of pancreatic islets of each size (β-cell area, mm^2^) was shown as a percentage of the total number. (**C**) The same analyses on 12- or 20–22-week-old mice are shown as in (**B**) (*n* = 4).

Recently, we constructed a mouse model by specifically deleting the phogrin gene in pancreatic β-cells, through a traditional Cre/LoxP conditional knockout system expressing the Cre recombinase driven by the rat insulin promoter, *RIP*, and showed that insulin content in βKO (RIP-Cre^+/−^ Phogrin^flox/flox^, βKO) islets was slightly less than that in control (RIP-Cre^+/−^ Phogrin^+/+^, Ctrl) islets [8,29]. This was due to a slight decrease in insulin granule density, as observed via electron microscopy [29]. However, blood insulin levels and insulin secretion from isolated islets upon high glucose stimulation were unaffected by phogrin deletion, whereas basal insulin release was slightly upregulated in βKO islets (Supplementary Figure S1). We have previously reported a reduced [^3^H]-thymidine incorporation rate in phogrin-deficient islets, possibly due to the specific regulation of IRS2 protein stability by phogrin [8]. Phogrin binds to IR via GSIS and stabilizes IRS2 levels by maintaining PTP1B activity, which contributes to β-cell growth. Therefore, we analyzed β-cell development in the pancreas by measuring β-cell mass. Seven-week-old control and βKO mice had similar β-cell mass per pancreas as assessed via immunostaining with insulin antibody (Figure 1A), and identical results were obtained when using adult mice (not shown). Since large islets significantly impact total β-cell mass, despite their small number, the islet size (β-cell area) in each mouse was sorted and the number of islets was distributed (Figure 1B). A resulting graph indicates no significant difference in the distribution between control and βKO. Similar results were obtained in adult mice (12- and 20–22-week), a very slight reduction in larger islets was observed in 20–22-week-old, phogrin-deficient mice (Figure 1C).

### 3.2. Phogrin Regulates High-Fat Diet-Induced Compensatory β-Cell Growth

**Figure 2:**
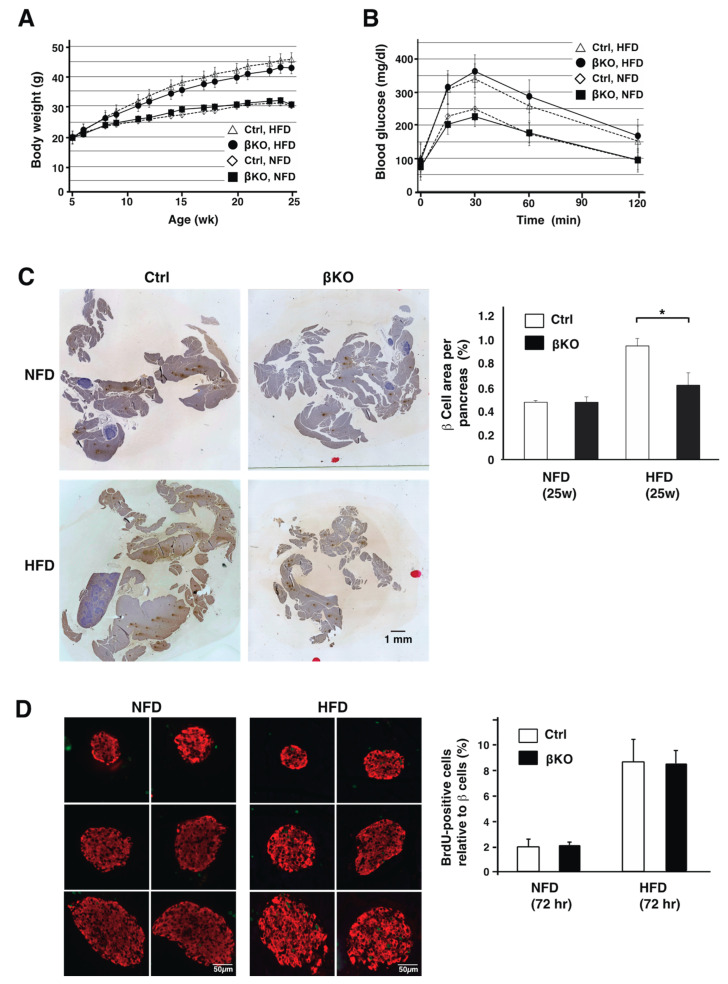
**Reduced compensatory β-cell expansion in HFD-fed, phogrin-knockout (βKO) mice:** (**A**) Body weight in Ctrl or βKO mice on a normal diet (NFD) or a high-fat. high-sucrose diet (HFD). Data are mean ± standard errors of the mean (SEM) from 7–31 male mice per group. (**B**) Glucose tolerance in 22-week-old Ctrl or βKO mice after 17 weeks on NFD or HFD. Data are means ± SEM from 13–25 male mice per group. (**C**) Immunohistochemical analyses of pancreas tissue from NFD- or HFD-fed Ctrl or βKO mice after 20 weeks using anti-insulin antibody. Each image is one of five composed sections for each mouse. Areas of β-cells relative to pancreas area were quantified and data are presented as means ± SEM (*n* = 4, * *p* < 0.05). (**D**) β-cell replication measured using a BrdU incorporation assay on NFD- or HFD-fed Ctrl or βKO mice after 3 days. Images are various sized islets from βKO mice fed a NFD (mouse No. 215) or HFD (No. 216). Results are shown as a ratio of BrdU-positive cells (green) to insulin-positive cells (red). Data are presented as means ± SEM (*n* = 4).

Since low-glucose, stress-induced apoptosis is increased in primary β-cells derived from phogrin-deficient mice [8], we examined the β-cell response in mice after high-fat, high-sucrose, diet (HFD)-induced metabolic stress. The body weight and glucose tolerance of HFD-fed control and βKO mice were not significantly different at 5–25 weeks (Figure 2A,B). Nevertheless, β-cell mass was indistinguishable between normal chow-fed control and βKO mice, but HFD-fed βKO mice had significantly lower compensatory β-cell hyperplasia than control mice (Figure 2C). Recent studies have reported that HFD-induced β-cell proliferation is prominent during the first week of feeding [30,31]. Thus, we assessed β-cell replication by measuring BrdU incorporation into insulin-containing β-cells. After 3 days, the BrdU incorporation rate increased in HFD-fed mice compared with that in normal chow-fed mice, and βKO mice showed a similar high β-cell replication rate in response to the HFD (Figure 2D). These results suggest that phogrin contributes to HFD-induced compensatory β-cell expansion but not early-response β-cell replication.

### 3.3. Interaction of Phogrin with IR Was Detectable in HFD-Fed Mice Islets

**Figure 3:**
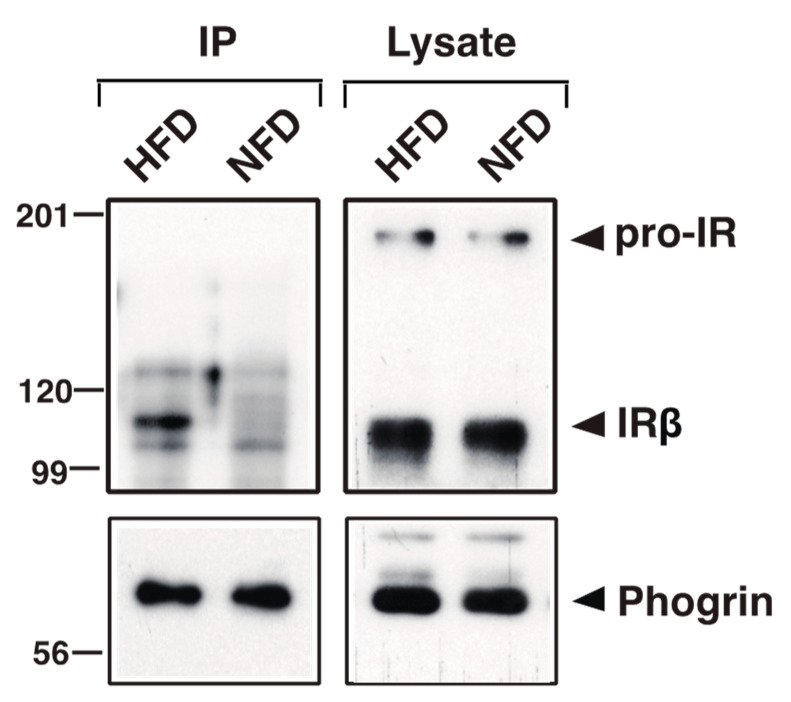
**Phogrin interacts with insulin receptors (IRs) in islets from high-fat, high-sucrose diet (HFD)-fed mice.** Islets isolated from wild-type mice fed a normal diet (NFD) or HFD were lysed, and 0.5 mg of each extract were immunoprecipitated with anti-phogrin rabbit antibody. Each immunoprecipitate and an original lysate aliquot (5 μg) were analyzed via immunoblotting with antibodies against insulin receptor β-subunit (IRβ) (upper panel). Phogrin amounts in each precipitate (5%) and lysate were determined via immunoblotting with anti-phogrin murine antibody (lower panels).

Recently, we have shown that phogrin-induced β-cell proliferation depends on its interaction with IR [19]. We, thus, evaluated whether HFD-induced β-cell expansion is associated with the phogrin/IR interaction. We prepared protein extracts from normal chow- or HFD-fed 19–23-week-old murine pancreatic islets and used them for co-immunoprecipitation with an anti-phogrin antibody. Notably, significantly more IRβ co-precipitated with phogrin from the HFD sample relative to the normal murine sample, whereas the phogrin and IR expression levels were constant (Figure 3). These results suggest that phogrin/IR-regulated β-cell growth mainly occurs in HFD-induced, metabolic-stress-exposed mice.

### 3.4. Intercellular Binding of Mature Phogrin Proteins Inhibits Phogrin/IR Complex Formation

**Figure 4:**
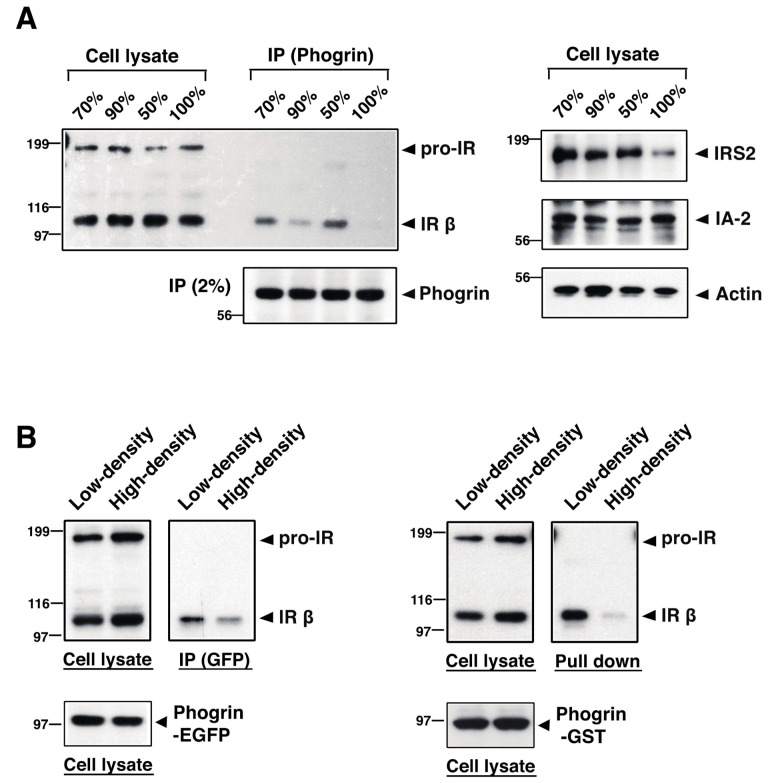
**Intracellular binding of phogrin to IR is attenuated in high-density conditions:** (**A**) MIN6 cells cultured at various cell densities (50–100%) for at least 24 h were extracted and an equal amount of each extract (1.5 mg) was immunoprecipitated with anti-phogrin antibody. The insulin receptor (IR) amount in each precipitate was determined via immunoblotting (left upper panel) and the immunoprecipitated phogrin level was also determined (left lower panel). IRS2, IA-2, and β-actin expression levels in each cell extract were analyzed via immunoblotting (right panels). (**B**) MIN6 cells expressing phogrin–EGFP (left panels) or phogrin–GST (right panels) were cultured at 90% (high) or 50% (low) cell density. Cell extracts were either immunoprecipitated with anti-GFP monoclonal antibody or pulled down with glutathione sepharose. The amount of IR in each precipitate and IR and phogrin expression levels in each lysate were determined via immunoblotting.

To clarify a condition of phogrin and IR complex formation, we examined the interaction rate of phogrin and IR using mouse β-cell line MIN6. Co-immunoprecipitation analysis showed that the phogrin/IR interaction rate was relatively low in cells cultured at high density (90–100%) but increased in growing (50–90%) cells (Figure 4A, left panel). The tyrosine phosphorylation levels of IRβ and IRS2 in cells cultured at a low density were also higher than those in high-density cells (Supplementary Figure S2A). IRS2 protein levels showed a similar tendency; namely, they were relatively high in growing MIN6 cells but low in cells cultured in dense conditions (Figure 4A, right panel). These results are consistent with data of previous studies, in which phogrin protected the IRS2 protein from degradation via direct binding to IR [8,19]. Next, we established two distinct stable cell lines expressing either phogrin–EGFP or phogrin–GST and confirmed the specific interaction of phogrin and IR via co-immunoprecipitation with an anti-GFP antibody or a pull-down assay with glutathione sepharose-beads, respectively (Supplementary Figure S2B). The cell density-dependent dissociation of phogrin and IR was similarly observed for these cells (Figure 4B).

**Figure 5:**
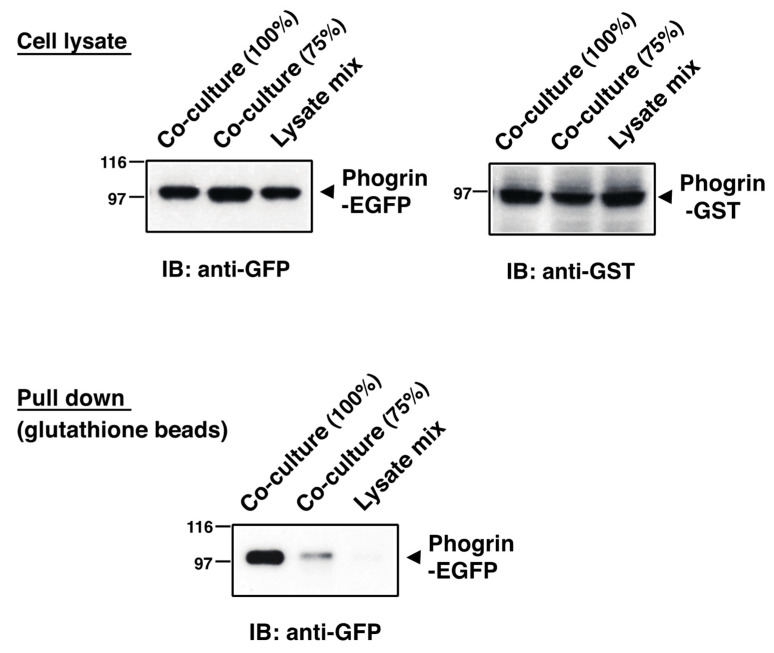
**Intercellular interaction of phogrin occurs in high-density co-cultures:** Cell extracts (2.0 mg protein) from MIN6/phogrin–EGFP and MIN6/phogrin–GST cultured together at high (100%) or low (75%) density for 48 h or extract mixtures (2.0 mg) from cells cultured separately were pulled down using glutathione sepharose. The presence of phogrin–EGFP in each precipitate (lower panel) and expression levels of phogrin–EGFP and phogrin–GST in each lysate (upper panels) were determined via immunoblotting.

Previous structural analyses have proposed that the matN domain (the cell surface region) of IA-2 (but not phogrin) forms dimers [32,33]. Thus, considering the intercellular interaction of cell surface phogrin in cells under high-density conditions, we dissected the phogrin complexes in a co-culture of two stable cell lines, MIN6/phogrin–EGFP and MIN6/phogrin–GST. Immunoblot analyses of protein extracts prepared from the co-cultured cells or the independently cultured cells confirmed the expression of similar amounts of phogrin–EGFP and phogrin–GST (Figure 5, upper panels). Notably, phogrin–EGFP was significantly detected in precipitates with glutathione beads from lysates of confluent cells (100%), whereas very small amounts of phogrin–EGFP or no signal were seen in lysates from subconfluent cells (75%) or mixtures of each cell lysate, respectively (Figure 5, lower panel). Taken together, these data suggest that the intercellular homologous binding of phogrin proteins at high cell densities inhibits the intracellular interaction between IR and phogrin.

## 4. Discussion

Phogrin and IA-2 constitute an evolutionarily conserved protein tyrosine phosphatase family that may play specific roles in neuroendocrine cells [1,2,3,4,5,6,7,8]. A series of analyses of the family genes have focused on pancreatic β-cells as both human corresponding proteins are autoantigens of human type 1 diabetes; however, their physiological roles and molecular functions remain undefined. In β-cell-specific, phogrin-knockout mice, no significant decrease in β-cell mass was observed via histochemical analysis, although phogrin silencing caused a marked retardation in cell growth in cultured cell lines [8,19]. Although the phogrin paralog IA-2 is thought to compensate for the loss of phogrin function, in our assays, IA-2 expression in phogrin-deficient islets was significantly decreased rather than increased [8]. In this study, we showed a condition under which phogrin regulates glucose-responsive proliferation in pancreatic β-cells.

We have previously shown that phogrin knockdown in β-cell lines retards cell growth with a specific decrease in IRS2 protein [8,19]. Proteasomal degradation reduces IRS2 in a time-delayed manner via a negative feedback mechanism of excess sustained signals. Hence, phogrin protects IRS2 and supports growth-promoting insulin signaling through transient binding to IR (or ternary interaction with IR and PTP1B). However, recent ex vivo studies in primary islets suggest that glucose-induced β-cell proliferation involves the IRS2–cyclin D2 pathway and carbohydrate-response element-binding protein (ChREBP)–heparin-binding EGF-like growth factor (HB-EGF) signaling rather than IR signaling [34,35]. These pathways are expected to involve complex signaling pathways such as IR downstream signals. Therefore, depending on the cellular milieu, pancreatic β-cells may use multiple associated pathways to promote glucose-induced cell proliferation. Our previous study showed that IRS2 protein expression in phogrin-deficient islets decreased at most mouse ages compared to that in control islets [8]; however, phogrin–IR binding, another marker of this function, could not be detected under normal conditions (Figure 3). Because β-cell proliferation signals in islets are generally weak under normal conditions, we considered the possibility that phogrin binds to IR under HFD conditions, which can induce β-cell proliferation. Consequently, co-immunoprecipitation with islet extracts from HFD-fed mice revealed the existence of the phogrin–IR complex (Figure 3).

Interestingly, the β-cell mass significantly reduced in HFD-fed phogrin KO mice at 20 weeks (Figure 2C), whereas the BrdU incorporation rate in the beginning three days was similar. These results suggested that phogrin was involved in compensatory β-cell proliferation in response to peripheral insulin resistance. This is consistent with the results of previous studies showing IRS2 involvement in insulin resistance–responsive compensatory β-cell proliferation [21]. Moreover, our data with the previous finding that compensatory β-cell proliferation in mice requires glycolysis and membrane depolarization [22] suggest that insulin secreted by glucose may act in an autocrine manner to promote β-cell growth when hyperglycemia is caused by HFD-induced insulin resistance. However, RIP-Cre transgenic mice should be treated with caution because Cre recombinase is ectopically expressed in the hypothalamus, and RIP-Cre mice themselves exhibit mild glucose intolerance [36,37]. Nevertheless, the lower proliferative activity in βKO mice relative to that in Ctrl mice, as detected in this study, is an important finding as there is no difference in insulin secretion or glucose tolerance between them (Figure 2B and Supplementary Figure S1). On the contrary, β-cell proliferation also decreases in systemic IA-2 gene knockout mice after pancreatectomy [20], suggesting that phogrin and IA2 may function for β-cell growth or restoration under metabolic stresses.

**Figure 6:**
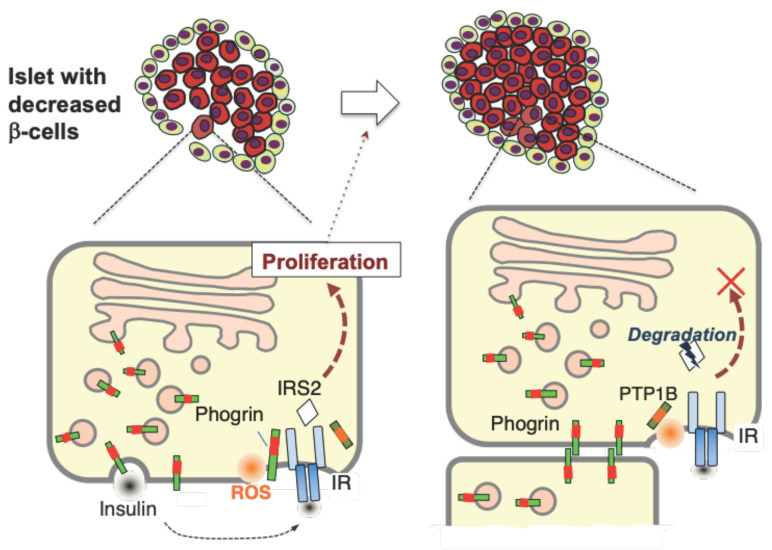
**A model for phogrin-mediated regulation of β-cell compensative proliferation.** Insulin resistance progression leads to β-cell damage or shedding, resulting in a transient β-cell decrease in the islets. Phogrin binds to IR on the plasma membrane and induces autocrine insulin signaling via IRS2 protein; consequently, β-cells proliferate and form a regular islet. Intercellular phogrin binding occurs at high cell density state and irreversible PTP1B inactivation induces IRS2 degradation, hence the proliferative signal stops. ROS, reactive oxygen species.

The proliferative regulation of phogrin in β-cells seems to express only under stress conditions. We identified the mechanism by which phogrin ceases to act on IR-mediated signaling. We first noticed a low phogrin and IR interaction rate in lysates from high-density MIN6 cells, which was elevated in growing MIN6 cells (lower density), with concomitant high expression of IRS2 (Figure 4). Proteomic analyses of pull-down experiments using normal murine islets indicated that mature phogrin is a candidate binding molecule. Consequently, our specific pull-down assay using the lysate from the co-culture of stable phogrin–EGFP- and phogrin–GST-expressing cells revealed a novel complex in which cell surface phogrin binds to the opposite side of phogrin instead of IR on the same membrane (Figure 5). Based on these results, we propose a functional model of β-cell phogrin within pancreatic islets (Figure 6). During the normal conditions such as development, the programmed β-cell proliferation occurs in the absence of phogrin-mediated autocrine insulin signaling. When mature β-cells are partially deleted by metabolic stress-driven apoptosis, phogrin–IR interaction induces the IRS2-mediated β-cell growth until β-cell mass is fully expanded. Finally, intercellular phogrin–phogrin interactions inhibit the associated autocrine insulin signaling when and where islet structures are properly built.

## 5. Conclusions

Our data show that the insulin granule protein phogrin is involved in HFD-associated compensatory β-cell growth in mice. Moreover, our data suggest that a population of proliferative β-cells appear in such conditions while most β-cells are stable in basal. In recent years, heterogeneity in adult β-cells has been examined using new techniques such as single cell analysis [27,28]. Although the relationship between phogrin expression and the proliferative potential of β-cells is undefined, immunostaining of murine islets with anti-phogrin antibodies detected the heterogeneous expression intensity. Further investigation could identify the exact location of β-cells responsible for compensatory proliferation in the mantle structure of the pancreatic islets.

## Data Availability

Data are contained within the article and supplementary materials.

## Supplementary Materials

The following supporting information can be downloaded at: https://www.mdpi.com/article/10.3390/nu16010169/s1. Figure S1: Insulin secretion is maintained in phogrin-deficient mice; Figure S2: Phogrin–IR complex exists in growing MIN6 and stable cell.

## References

[1] Wasmeier C., Hutton J.C. (1996). Molecular cloning of phogrin, a protein-tyrosine phosphatase homologue localized to insulin secretory granule membranes. J. Biol. Chem..

[2] Lu J., Li Q., Xie H., Chen Z.J., Borovitskaya A.E., Maclaren N.K., Notkins A.L., Lan M.S. (1996). Identification of a second transmembrane protein tyrosine phosphatase, IA-2beta, as an autoantigen in insulin-dependent diabetes mellitus: Precursor of the 37-kDa tryptic fragment. Proc. Natl. Acad. Sci. USA.

[3] Cui L., Yu W., DeAizpurua H.J., Schmidli R.S., Pallen C.J. (1996). Cloning and characterization of islet cell antigen-related protein-tyrosine phosphatase (PTP), a novel receptor-like PTP and autoantigen in insulin-dependent diabetes. J. Biol. Chem..

[4] Chiang M.K., Flanagan J.G. (1996). PTP-NP, a new member of the receptor protein tyrosine phosphatase family, implicated in development of nervous system and pancreatic endocrine cells. Development.

[5] Solimena M., Dirkx R., Hermel J.M., Pleasic-Williams S., A Shapiro J., Caron L., Rabin D.U. (1996). ICA 512, an autoantigen of type I diabetes, is an intrinsic membrane protein of neurosecretory granules. EMBO J..

[6] Fitzgerald L.R., Walton K.M., Dixon J.E., Largent B.L. (1997). PTP NE-6: A brain-enriched receptor-type protein tyrosine phosphatase with a divergent catalytic domain. J. Neurochem..

[7] Drake P.G., Peters G.H., Andersen H.S., Hendriks W., Moller N.P. (2003). A novel strategy for the development of selective active-site inhibitors of the protein tyrosine phosphatase-like proteins islet-cell antigen 512 (IA-2) and phogrin (IA-2beta). Biochem. J..

[8] Torii S., Kubota C., Saito N., Kawano A., Hou N., Kobayashi M., Torii R., Hosaka M., Kitamura T., Takeuchi T. (2018). The pseudophosphatase phogrin enables glucose-stimulated insulin signaling in pancreatic β cells. J. Biol. Chem..

[9] Caromile L.A., Oganesian A., Coats S.A., Seifert R.A., Bowen-Pope D.F. (2010). The neurosecretory vesicle protein phogrin functions as a phosphatidylinositol phosphatase to regulate insulin secretion. J. Biol. Chem..

[10] Takeyama N., Ano Y., Wu G., Kubota N., Saeki K., Sakudo A., Momotani E., Sugiura K., Yukawa M., Onodera T. (2009). Localization of insulinoma associated protein 2, IA-2 in mouse neuroendocrine tissues using two novel monoclonal antibodies. Life Sci..

[11] Gomi H., Kubota-Murata C., Yasui T., Tsukise A., Torii S. (2013). Immunohistochemical analysis of IA-2 family of protein tyrosine phosphatases in rat gastrointestinal endocrine cells. J. Histochem. Cytochem..

[12] Ramírez-Franco J.J., Munoz-Cuevas F.J., Luján R., Jurado S. (2016). Excitatory and inhibitory neurons in the hippocampus exhibit molecularly distinct large dense core vesicles. Front. Cell Neurosci..

[13] Suckale J., Solimena M. (2010). The insulin secretory granule as a signaling hub. Trends Endocrinol. Metab..

[14] Omar-Hmeadi M., Idevall-Hagren O. (2021). Insulin granule biogenesis and exocytosis. Cell. Mol. Life Sci. CMLS.

[15] Saeki K., Zhu M., Kubosaki A., Xie J., Lan M.S., Notkins A.L. (2002). Targeted disruption of the protein tyrosine phosphatase-like molecule IA-2 results in alterations in glucose tolerance tests and insulin secretion. Diabetes.

[16] Kubosaki A., Gross S., Miura J., Saeki K., Zhu M., Nakamura S., Hendriks W., Notkins A.L. (2004). Targeted disruption of the IA-2beta gene causes glucose intolerance and impairs insulin secretion but does not prevent the development of diabetes in NOD mice. Diabetes.

[17] Henquin J.C., Nenquin M., Szollosi A., Kubosaki A., Notkins A.L. (2008). Insulin secretion in islets from mice with a double knockout for the dense core vesicle proteins islet antigen-2 (IA-2) and IA-2beta. J. Endocrinol..

[18] Cai T., Hirai H., Zhang G., Zhang M., Takahashi N., Kasai H., Satin L.S., Leapman R.D., Notkins A.L. (2011). Deletion of Ia-2 and/or Ia-2beta in mice decreases insulin secretion by reducing the number of dense core vesicles. Diabetologia.

[19] Torii S., Saito N., Kawano A., Hou N., Ueki K., Kulkarni R.N., Takeuchi T. (2009). Gene silencing of phogrin unveils its essential role in glucose-responsive pancreatic beta-cell growth. Diabetes.

[20] Mziaut H., Kersting S., Knoch K.P., Fan W.H., Trajkovski M., Erdmann K., Bergert H., Ehehalt F., Saeger H.D., Solimena M. (2008). ICA512 signaling enhances pancreatic beta-cell proliferation by regulating cyclins D through STATs. Proc. Natl. Acad. Sci. USA.

[21] Terauchi Y., Takamoto I., Kubota N., Matsui J., Suzuki R., Komeda K., Hara A., Toyoda Y., Miwa I., Aizawa S. (2007). Glucokinase and IRS-2 are required for compensatory beta cell hyperplasia in response to high-fat diet-induced insulin resistance. J. Clin. Investig..

[22] Porat S., Weinberg-Corem N., Tornovsky-Babaey S., Schyr-Ben-Haroush R., Hija A., Stolovich-Rain M., Dadon D., Granot Z., Ben-Hur V., White P. (2011). Control of pancreatic beta cell regeneration by glucose metabolism. Cell Metab..

[23] Rhodes C.J. (2005). Type 2 diabetes-a matter of beta-cell life and death?. Science.

[24] Kubota T., Kubota N., Kadowaki T. (2017). Imbalanced Insulin Actions in Obesity and Type 2 Diabetes: Key Mouse Models of Insulin Signaling Pathway. Cell Metab..

[25] Weir G.C., Gaglia J., Bonner-Weir S. (2020). Inadequate β-cell mass is essential for the pathogenesis of type 2 diabetes. Lancet Diabetes Endocrinol..

[26] Bader E., Migliorini A., Gegg M., Moruzzi N., Gerdes J., Roscioni S.S., Bakhti M., Brandl E., Irmler M., Beckers J. (2016). Identification of proliferative and mature β-cells in the islets of Langerhans. Nature.

[27] Wang Y.J., Kaestner K.H. (2019). Single-Cell RNA-Seq of the Pancreatic Islets—A Promise Not yet Fulfilled?. Cell Metab..

[28] Miranda M.A., Macias-Velasco J.F., Lawson H.A. (2021). Pancreatic β-cell heterogeneity in health and diabetes: Classes, sources, and subtypes. Am. J. Physiol. Endocrinol. Metab..

[29] Yasui T., Mashiko M., Obi A., Mori H., Ito-Murata M., Hayakawa H., Kikuchi S., Hosaka M., Kubota C., Torii S. (2023). Insulin granule morphology and crinosome formation in mice lacking the pancreatic β cell-specific phogrin (PTPRN2) gene. Histochem. Cell Biol..

[30] Stamateris R.E., Sharma R.B., Hollern D.A., Alonso L.C. (2013). Adaptive beta-cell proliferation increases early in high-fat feeding in mice, concurrent with metabolic changes, with induction of islet cyclin D2 expression. Am. J. Physiol. Endocrinol. Metab..

[31] Mosser R.E., Maulis M.F., Moulle V.S., Dunn J.C., Carboneau B.A., Arasi K., Pappan K., Poitout V., Gannon M. (2015). High-fat diet-induced beta-cell proliferation occurs prior to insulin resistance in C57Bl/6J male mice. Am. J. Physiol. Endocrinol. Metab..

[32] Primo M.E., Jakoncic J., Noguera M.E., Risso V.A., Sosa L., Sica M.P., Solimena M., Poskus E., Ermácora M.R. (2011). Protein-protein interactions in crystals of the human receptor-type protein tyrosine phosphatase ICA512 ectodomain. PLoS ONE.

[33] Noguera M.E., Primo M.E., Jakoncic J., Poskus E., Solimena M., Ermácora M.R. (2015). X-ray structure of the mature ectodomain of phogrin. J. Struct. Funct. Genom..

[34] Stamateris R.E., Sharma R.B., Kong Y., Ebrahimpour P., Panday D., Ranganath P., Zou B., Levitt H., Parambil N.A., O’Donnell C.P. (2016). Glucose Induces Mouse β-Cell Proliferation via IRS2, MTOR, and Cyclin D2 but Not the Insulin Receptor. Diabetes.

[35] Maachi H., Fergusson G., Ethier M., Brill G.N., Katz L.S., Honig L.B., Metukuri M.R., Scott D.K., Ghislain J., Poitout V. (2020). HB-EGF Signaling Is Required for Glucose-Induced Pancreatic β-Cell Proliferation in Rats. Diabetes.

[36] Lee J.Y., Ristow M., Lin X., White M.F., Magnuson M.A., Hennighausen L. (2006). RIP-Cre revisited, evidence for impairments of pancreatic beta-cell function. J. Biol. Chem..

[37] Song J., Xu Y., Hu X., Choi B., Tong Q. (2010). Brain expression of Cre recombinase driven by pancreas-specific promoters. Genesis.

