# Supplementary material for: Phogrin Regulates High-Fat Diet-Induced Compensatory Pancreatic β-Cell Growth by Switching Binding Partners
Source: Nutrients. 2024 Jan 4;16(1):169. doi: 10.3390/nu16010169 (PMC10780347; doi:10.3390/nu16010169)
Supplement: Supplementary file 1 [file nutrients-16-00169-s001.zip › nutrients-2783597-supplementary.pdf]

Supplementary Figure 1 Kubota et al

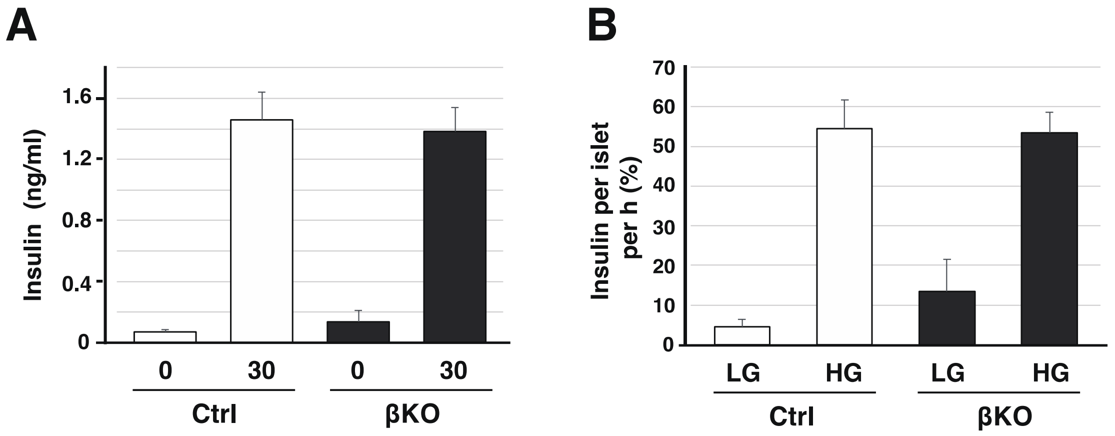

Figure S1. Insulin secretion is maintained in phogrin-deficient mice. (A) Insulin secretion profile of control (Ctrl: $Cre^{+/-}$_*Phogrin*$^{+/+}$) or knockout (βKO: $Cre^{+/-}$_*Phogrin*$^{fl/fl}$) 16-17-week-old male mice. The serum insulin from mice before (0) and after 30 min of glucose stimulation were analyzed by ELISA (n=6). (B) Pancreatic islets isolated from Ctrl or βKO mice were stimulated by low (2.2 mM) or high (17.8 mM) glucose, and then collected media were analyzed by ELISA (n=5). All data are presented as mean ± standard errors of the mean (SEM).

Supplementary Figure 2 Kubota et al

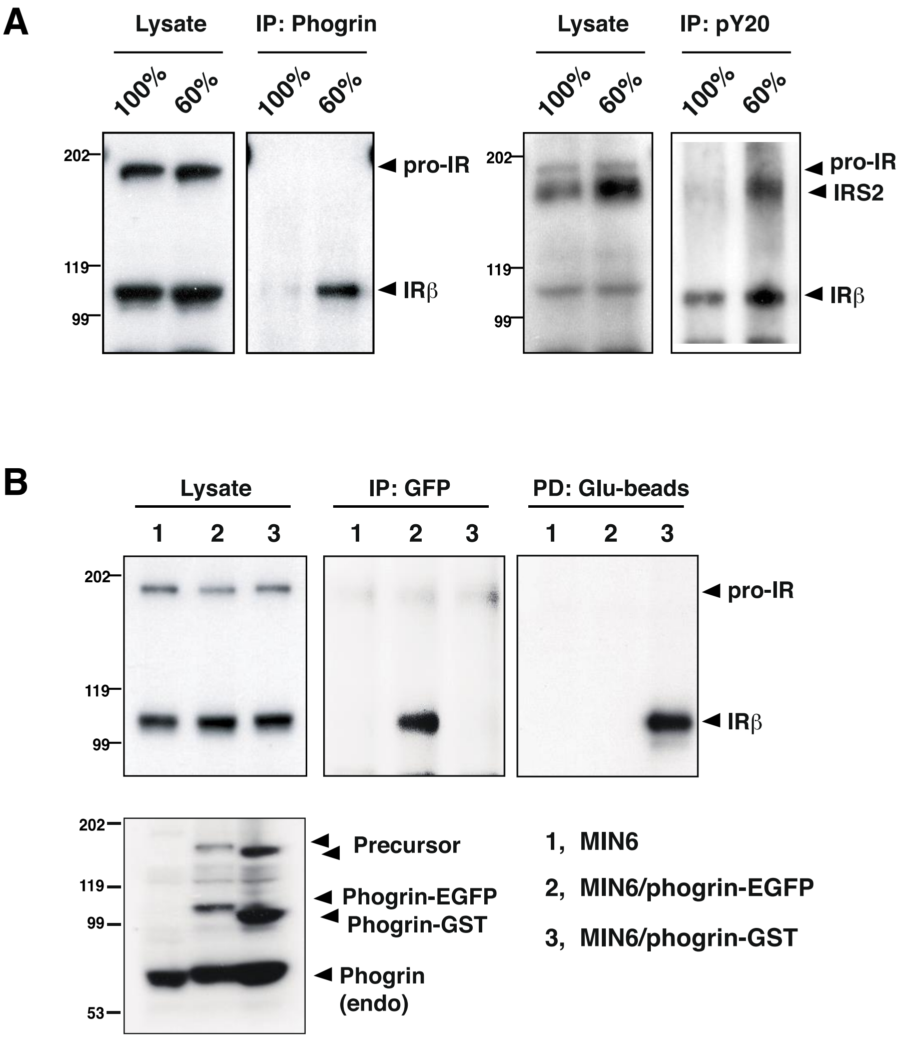

Figure S2. Phogrin–IR complex exists in growing MIN6 and stable cells. (A) MIN6 cells were cultured at 100% (High) or 60% (Low) cell density. Cell extracts were immunoprecipitated with anti-phogrin antibody or anti-phospho-tyrosine antibody (pY20). The amount of IR in phogrin-precipitate and the phosphorylation levels of IR and IRS2 were determined by immunoblotting. (B) MIN6 cells or MIN6 stable cells expressing phogrin–EGFP or phogrin–GST were cultured at low (75%) density for 48 h. Cell extracts were either immunoprecipitated with anti-GFP monoclonal antibody or pulled down with glutathione sepharose. The amount of IR in each precipitate and IR and phogrin expression levels in each lysate were determined by immunoblotting.
